# Exploring Laypersons’ Experiences With a Mobile Symptom Checker App as an Interface Between eHealth Literacy, Health Literacy, and Health-Related Behavior: Qualitative Interview Study

**DOI:** 10.2196/60647

**Published:** 2025-03-21

**Authors:** Roland Koch, Marie-Theres Steffen, Anna-Jasmin Wetzel, Christine Preiser, Malte Klemmt, Hans-Jörg Ehni, Regina Mueller, Stefanie Joos

**Affiliations:** 1 Institute for General Practice and Interprofessional Care Tübingen University Hospital Tübingen Germany; 2 Institute of Occupational and Social Medicine and Health Services Research University Hospital Tübingen Tübingen Germany; 3 Institute of Applied Social Science University of Applied Science Würzburg-Schweinfurt Würzburg Germany; 4 Institute for General Practice and Palliative Care Medizinische Hochschule Hannover Hanover Germany; 5 Institute of Ethics and History of Medicine University Hospital Tübingen Tübingen Germany; 6 Institute of Philosophy University of Bremen Bremen Germany

**Keywords:** symptom checker apps, health literacy, eHealth literacy, qualitative research, interview study, artificial intelligence, AI

## Abstract

**Background:**

Symptom checkers aim to help users recognize medical symptoms and recommend actions. However, they are not yet reliable for self-triage or diagnostics. Health literacy plays a role in their use, but the process from symptom recognition to health care consultation remains unclear.

**Objective:**

This qualitative observatory study explored how laypersons use symptom checkers, focusing on the process of use, entry points and outcomes, and the role of health literacy. Laypersons are defined as individuals who are neither medical professionals nor developers of such apps. Three research questions were addressed: (1) How do such users describe the process of using symptom checkers? (2) What are entry points and possible outcomes of symptom checker app use? (3) How are health literacy and eHealth literacy expressed during the use of symptom checker apps?

**Methods:**

As part of the Ethical, Legal, and Social Implications of Symptom Checker Apps in Primary Health Care project, 15 laypersons (n=9, 60% female and n=6, 40% male; mean age 30.7, SD 13.6 years) were interviewed about their experiences with the symptom checker Ada. The interviews were analyzed using an integrative approach combining social positioning, agency, and the Rubicon model as a heuristic framework.

**Results:**

App use follows a cyclic process comprising 4 steps: motivation (influenced by biography and context), intention formation (assigning a purpose), intention implementation (recruiting resources), and evaluation (transforming interactions into health-related insights). Biographical, social, and contextual factors shape process initiation. Users use symptom checkers for 3 main purposes: understanding their condition, receiving recommendations for action, and documenting or communicating health-related information. Each purpose requires specific planning and integration into health-related behaviors drawing on personal, social, and technological resources. Evaluation depends on contextual factors, app outputs, and the outcomes of users’ health-related actions. Users assess whether the app aligns with their expectations, condition severity, and previous experiences, with health literacy playing a critical role in validation processes.

**Conclusions:**

Symptom checker use is a complex, cyclic process shaped by context, biography, and health literacy. Users are motivated by health concerns influenced by personal, social, and contextual factors, with trust and attitudes impacting initial engagement. Intention formation reflects a balance between user skills and context, where app outputs inform decisions but may not always lead to action, especially in ambiguous situations. Users rely on personal resources and social networks to integrate app use into health-related behaviors, highlighting the limitations of symptom checkers in providing social or empathetic support. Symptom checkers have the potential to serve as an interface between users and health care, but future development must address the complexity of their use to unlock this potential.

**International Registered Report Identifier (IRRID):**

RR2-10.2196/34026

## Introduction

### Background

This qualitative study investigated how eHealth literacy (eHL) [1,2], health literacy (HL) [3-6], and health-related behavior (HRB) influence how laypersons, defined as individuals who are neither medical professionals nor developers of symptom checker apps, engage with a specific mobile symptom checker app. Symptom checker apps offer readily accessible tools for self-triage by prompting users to enter health-related information [3,7]. This study focused on the Ada app by Ada Health GmbH.

Research shows that symptom checker apps are often used out of curiosity or anxiety or to confirm the severity of symptoms [4,7-9]. Factors such as users’ age, confidence in self-assessment, trust in the app and technology, technology affinity, past experiences with the health care system, and proficiency in navigating health information play critical roles in shaping user experiences and engagement with symptom checker apps [3-7,9-16].

There is limited and partly conflicting evidence to conclude whether symptom checker app use directly prompts HRB, such as seeking professional health care [3,9,13,16,17]. Emerging literature suggests that symptom checker app use may foster self-care but could also lead to unnecessary health care use, particularly when the apps err on the side of caution in nonacute settings (eg, feeling tired over several days) [18-22]. Symptom checker app use can influence users’ anxiety about seeking medical help [3,4,17,23], with many preferring “wait and see” recommendations [7,17], although satisfaction with the app does not always correlate with adherence to its advice [13,24].

In addition, previous studies highlight that eHL and HL significantly impact users’ interaction with digital health tools and health outcomes, yet there is a knowledge gap in understanding these literacy concepts in the context of symptom checker app use [25-31]. Another gap in the literature is how the social context influences the use of digital health applications such as symptom checker apps, especially considering that eHL and HL are distributed unequally across regions and social groups [32-36].

How the occurrence of a symptom leads layperson users to engage in symptom checker app use and, consecutively, act on the app’s output has not yet been described as a process [3,37]. There are several challenges to researching symptom checker apps, such as different user profiles, the context dependency of app use, and the rapid rate of development in the field [3,14,38].

### Objectives

This study aimed to fill that gap by describing the process through which layperson users engage with symptom checker apps for health concerns, examining the influence of eHL and HRB on their interactions with the app and possible outcomes. Specifically, this study addressed (1) the steps that layperson users follow in using symptom checker apps, (2) key entry points and outcomes of symptom checker app use, and (3) the roles of eHL and HL in this process.

## Methods

### Study Design

This observational, exploratory qualitative study was embedded in the larger context of the joint Ethical, Legal, and Social Implications of Symptom Checker Apps in Primary Health Care (CHECK.APP) project [39]. CHECK.APP represents a collaboration among the Institute for General Practice and Interprofessional Care, the Institute of Ethics and History of Medicine, the Institute for Occupational and Social Medicine and Health Services Research at Tübingen University Hospital, and the Institute of Applied Social Science. The interdisciplinary research team consists of persons with professional training and research experience in medicine, family medicine, psychology, sociology, law, medical ethics, and philosophy. The project is conducted in the context of the German health care system with a focus on primary health care.

The CHECK.APP project is based on a mixed methods design and has four foci: (1) ethical, legal, and social issues [3]; (2) epidemiology of symptom checker app use and predictors of use [9]; (3) patterns of symptom checker app use and impact on individuals [16]; and (4) impact of symptom checker app use on the health care system and health care workers [40]. This study was part of focus 3. Focus 3 consisted of a diary study that was complemented with a follow-up qualitative interview study. The 6-week self-monitoring diary study aimed to generate mixed methods data on symptom checker app use patterns [16].

This study was conducted following the diary study and was designed to complement the quantitative analysis of the use patterns observed in the diary study by providing an in-depth exploration of the motivations and experiences of symptom checker app users. The reporting of this study followed the Standards for Reporting Qualitative Research guidelines statement [41]. The reporting checklist is included as Multimedia Appendix 1.

### How Ada Works

On the Ada app, users enter their symptoms by responding to a series of questions. On the basis of their answers, Ada generates follow-up questions to refine the symptom description. For example, if a user reports a rash, the app displays various images of rashes, prompting the user to select the one that best matches their condition. The app then yields a list of possible causes, recommendations on care urgency, and self-care advice (eg, “Eight of ten users with these symptoms had an acute infection—it is recommended that you go see a doctor”).

### Sample

For sample size estimation, we applied the 5D model of information power by Malterud et al [42], which considers study aim, sample specificity, use of established theory, quality of dialogue, and analysis strategy. According to the 5D model, the more specific each dimension, the smaller the required sample size in a qualitative study. Given the study aim of an in-depth exploration of symptom checker app use by laypersons, the specific focus on users of a single app, the analysis approach grounded in established theory (see the Data Analysis section), the expertise of the researchers involved with an expected high dialogue quality, and the planned techniques to enhance trustworthiness (see the Data Analysis section), a sample of 15 interviews was estimated to generate sufficient information power to explore the phenomenon under study. We reserved the option to recruit additional participants if the estimated sample size proved insufficient. To determine this, the analysis began with an initial reading of the transcripts even before the interviews were completed (see the Data Analysis section).

Participants for this study were recruited from a pool of 48 active symptom checker app users who had previously completed the 6-week self-observation diary study. All participants in the diary study were active users of Ada (Ada Health GmbH) and were originally recruited through the survey conducted as part of the CHECK.APP project. Recruitment was limited to existing Ada users to comply with ethical requirements prohibiting the initiation of symptom checker app use for the study. Participants used Ada within the context of their daily lives. Incentives were provided for maintaining the diary and participating in the interviews.

These diary study participants were approached and asked whether they were interested in undergoing additional in-depth interviews based on their respective diary experiences. All 48 persons who took part in the diary study were laypersons, meaning that they were neither health care professionals nor symptom checker app developers [16]. A chronic condition was reported by 21% (10/48) of the potential interview partners. In total, 35% (17/48) of the potential participants used the symptom checker app infrequently (less than once per week). Mean age was 27 (SD 9.1; range 19-64) years. A total of 65% (31/48) of the potential participants identified as female, and 35% (17/48) identified as male. No person identified as nonbinary. Racial characteristics were not asked about in the diary study [16]. Of the 48 persons who took part in the diary study, 28 (58%) expressed interest in an interview. The participants of the diary study from whom the interviewees were selected primarily consisted of well-educated younger female adults, a limitation discussed later in the manuscript.

Among these 28 persons, interviewees were stratified based on age, gender, use frequency, and experiences addressed in the diaries to achieve the best heterogeneity within the rather homogeneous sample. The relevant characteristics of the 15 selected interview participants can be found in Table 1. Mean age was 30.7 (SD 13.6) years.

**Table 1:** Study sample for the symptom checker app user interviews (N=15).

Relative dimension and features		Participants, n (%)
**Age (y)**		
	20-29	10 (67)
	30-39	2 (13)
	40-49	1 (7)
	50-59	1 (7)
	60-69	1 (7)
**Gender**		
	Women	9 (60)
	Men	6 (40)
	Diverse or nonbinary	0 (0)^a^
**Symptom checker app use frequency**		
	Sparse (less than once per week)	6 (40)
	Frequent (more than once per week)	9 (60)
**Educational level**		
	Upper secondary school leading to university entrance qualification	12 (80)
	Intermediate secondary school	2 (13)
	Basic secondary school	1 (7)
**Place of residence**		
	Urban	13 (87)
	Rural	2 (13)
**Chronic condition**		
	Present	5 (33)
	Not present	10 (67)

^a^Despite our efforts, no nonbinary or diverse persons were included in the study.

### Data Collection

The interviews were conducted by a pair of interviewers using an interview guide (Multimedia Appendix 2). The interview guide was created by the research team for this study and piloted with 2 interview partners who were not included in the analysis. The test interviews were used to review the guide and interview styles in a group feedback discussion between the interviewers and their supervisors. No changes were made to the interview guide during data collection. The interviews were conducted by a pair of interviewers comprising 1 representative from the Institute for General Practice and Interprofessional Care (AJW, RK, or MTS) and 1 representative from either the Institute of Ethics and History of Medicine (RM) or the Institute of Applied Social Science (MK). After consent was obtained, the interviews were conducted using the Zoom (Zoom Video Communications) video client and audio recorded on local, secure computers at Tübingen University Hospital. The interviews were conducted from January 18, 2022, to March 15, 2022, with an average duration of 46 (SD 10.4; range 35-76) minutes.

The interviews were transcribed pseudonymously by a certified office (Amanu). The transcripts were stored on protected servers at Tübingen University Hospital. For analysis, Microsoft Excel and Microsoft Word (Microsoft Corp) were used.

### Data Analysis

This qualitative interview study followed a constructivist research paradigm—users construct their social reality by integrating experiences, self-image, daily interactions, and bodily perceptions into their biography [43]. They learn from experience [44]. These integrated factors are expressed through the social act of narration during the interviews.

**Figure 1:**
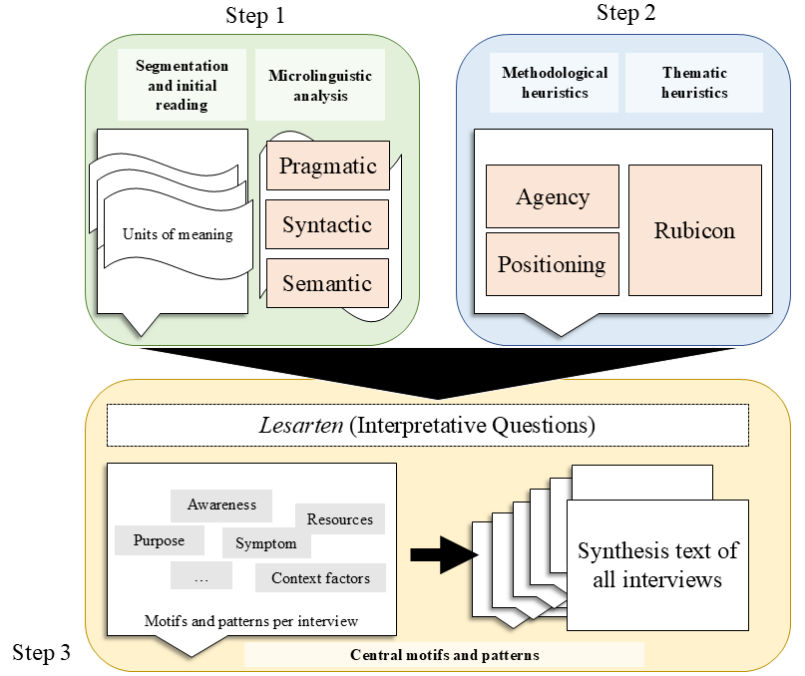
Visual representation of the integrative basic method. The process begins with 2 analysis steps: segmentation into units of meaning and microlinguistic analysis (step 1) and methodological and thematic heuristics, which are applied to the units of meaning (step 2). Both steps are then unified through interpretative questions (“Lesarten”) into a final step (step 3), identifying central motifs and patterns within and across the interviews. Speech bubbles symbolize interviews, whereas flags denote units of meaning.

The integrative basic method by Kruse [45] is an interpretative-reconstructive qualitative approach to reveal manifest and latent meanings and the way in which users “make sense” of the app and their experience with it. It represents an in-depth analysis of self-perception, biographical connections, self-image, and the perception of social roles within interview material [45]. The analysis was conducted by RK and MTS using strategies to enhance trustworthiness and rigor (see the Data Analysis section). The process is visualized in Figure 1.

Initial transcript reading commenced on March 1, 2022, while 3 interviews were still pending. This revealed thematic complexity; distinct patterns; and notable contrasts, contradictions, and differences within the data. As a result, recruitment of additional participants beyond the planned sample size of 15 interviews was deemed unnecessary.

The first step in the integrative basic method is a detailed microlinguistic analysis in which transcripts are segmented into discrete units of meaning guided by cues such as pauses, interviewer questions, and topic changes. Each unit is then analyzed on three distinct linguistic levels capturing different aspects of the speaker’s attention: (1) pragmatic (interactional aspects, including the relationship between interviewer and participant), (2) syntactic (grammatical choices that reflect cognitive structures), and (3) semantic (specific word choices, metaphors, and other lexical elements). This descriptive in-depth analysis of interaction, syntax, and semantics lays the groundwork for the following analysis of concepts such as agency, positioning, and the Rubicon model. Microsoft Excel was used for this step.

The transcripts were then analyzed using methodological and thematic heuristics. In our case, an analysis of social positioning and agency was used to understand how layperson users perceive the symptom checker app themselves and their scope for action embedded in a network of participants [46,47]. In terms of thematic heuristics, we used the Rubicon model by Achtziger and Gollwitzer [48] as a framework for motivational processes and intention building. It served both as a thematic lens to help explore the intrapersonal motivational process and as a macrostructure for the final synthesis text. The analyses were conducted using Microsoft Word.

In the final stage, the preceding microlinguistic analysis and the methodological and thematic analyses were condensed and compared against the background of the research questions to identify core motifs and modes of thematization within each interview and between interviews. This step began with formulating interpretative questions (“Lesarten” in German). The questions formed critical bridges between microanalysis (step 1) and interpretative analysis (step 2) to ensure that the interpretations remained grounded in the data. For instance, a question might be the following: “What role does Ada play in the progression from symptom onset to the utilization of health care?”

By answering the questions, central motifs and patterns specific to each interview were identified inductively. These motifs and patterns were then compared across all interviews to identify recurring themes (commonalities) and deviations within and across transcripts. While commonalities were used to generalize the findings, deviations were used to refine them. These insights were then used to reveal and formulate central, overarching motifs and patterns that were categorized using headlines (eg, “Context of app use”) and structured according to the steps of the Rubicon model. Microsoft Excel was used for this step.

To enhance trustworthiness, RK and MTS performed each of the aforementioned 3 analysis steps independently on the same transcript. They then compared their results after each step. Differences were discussed, and if possible, a mutual understanding of the texts and the method was established. When disagreements arose, they were discussed in monthly multidisciplinary methodological workshops within the CHECK.APP project research team.

Relying on the method of peer checking, the interim results were presented and discussed several times in a multidisciplinary research workshop on qualitative research methods. For a member check with most of the interview partners, the interim results were discussed and validated during a 3-hour session. Furthermore, in a multidisciplinary validation workshop, intermediate states of the analysis as well as open questions were discussed and illuminated under consideration of the perspectives of law, ethics, social medicine, and general medicine within the CHECK.APP project team. The results of these discussions were used to inform interpretative analysis (eg, by considering more perspectives) and the formulation of the final results text.

### Ethical Considerations

This study received a positive vote from the ethics committee of the University of Tübingen (464/2020BO) and was conducted following the Declaration of Helsinki. All participants gave their informed consent before the interviews began. The participants received monetary compensation (€50, approximately US $50) for taking part in the interviews. All data were stored on encrypted servers.

## Results

### Visualization of the Symptom Checker App Use Process

**Figure 2:**
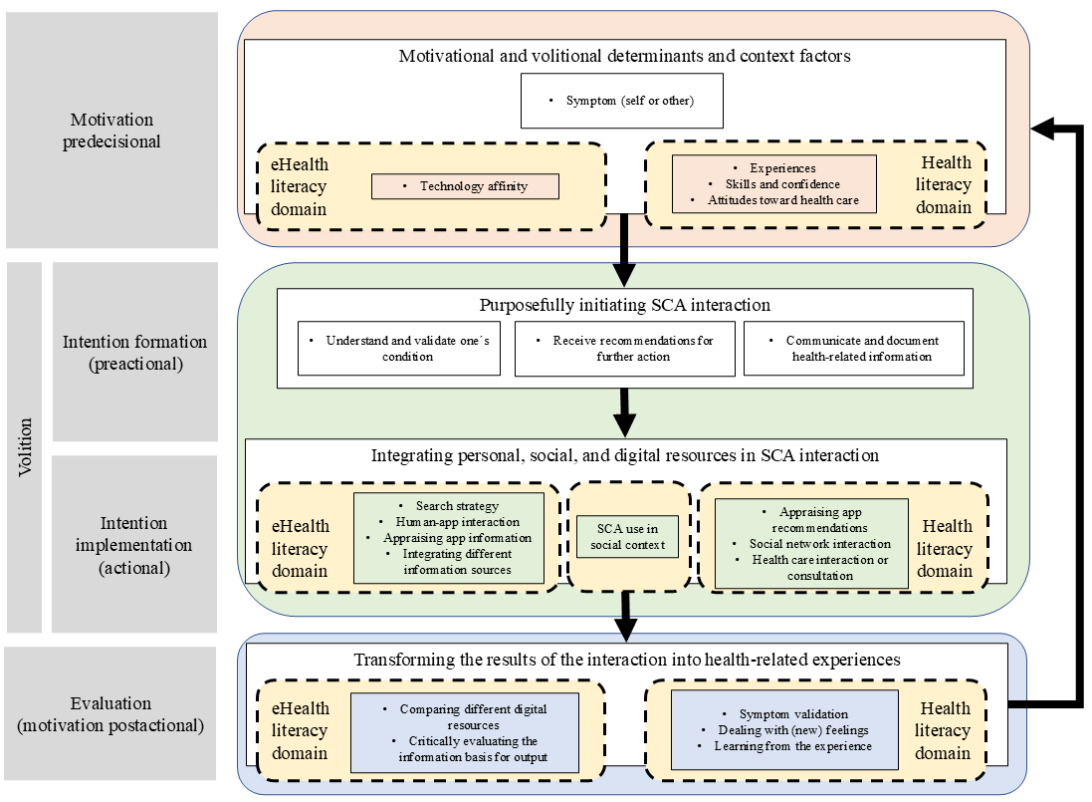
Symptom checker app (SCA) use process based on 15 interviews with SCA users. The figure is based on the Rubicon model. It describes the path from an entry point (such as noticing a health-related concern) via intention formation (influenced by motivational and volitional determinants) and volition to health-related behavior as an outcome. The latter can take place in different contexts. The behavior, in turn, leads to new experiences that are evaluated by the individual and integrated into the biography.

On the basis of the 4 stages of the Rubicon model, a cyclic model of the symptom checker app use process according to the interview partners was developed. It is presented in Figure 2.

In the following sections, the synthesized text produced through the integrative basic method is presented. Interview excerpts are provided to illustrate the data basis for the central motifs and patterns. Unless stated otherwise, the excerpts are from the interview partners. If the interviewer asked a question, it is marked with “interviewer.”

### Motivation: Predecisional

#### Motivational Stage

Users assessed the severity of the issue by comparing it to their own preexisting experiences. The interview partners did not reflect on the relevance or validity of their perceived symptoms and experiences—they took for granted that both were real and valid. If the symptoms were perceived as less severe and comparable experiences were available in the users’ biography, the sensation was described as manageable:

Yes. I have that once in a while. I don’t even know where that comes from. I also think that it’s possibly one of those things that kind of comes from stress, I definitely feel like that. And because I just never knew what it was, but I also knew that it would go away again, so because I had it before, as I said, I just looked it up in the app once.

NT45

At times, when individuals could not find relatable experiences from their own health history or perceived the issue as more severe, they expressed negative emotions such as fear or shock:

...In the end, it was a migraine attack. But I didn’t think about it at that moment, I was shocked at first.

NT39

Trust in the app also played a role in the decision to use it, as the following excerpt illustrates. The same interview partner continued to elaborate on how trust in the app competed with trust in health care professionals:

But, yes, because I couldn’t describe my problem in detail—I didn’t trust the app so much at that moment—it was important for me to go to the doctor quickly, because I knew, okay, there are important things that I couldn’t tell the app, and then that would explain the diagnosis, so to speak.

NT41

#### Context of App Use

Interview partners reported on different contexts in which they considered using the app. The most commonly reported context was the occurrence of a health-related concern. They first reflected on whether the symptom was even “real”—meaning whether they had just imagined it or whether the perceptions really represented an issue worth dealing with. They compared the symptom with their health-related experiences and biography and tried to make sense of what was happening to them:

Yes, I also clearly had chest pains. I mean, at that point I was already asking myself: Okay, to what extent is this happening now? But I mean, then I lay there and I had the feeling and thought: Yes, come on—so it was already real for me at that moment.

NT47

Users also reported being motivated to use the app by the health-related concerns of others. One user reported that she used the app for her family. She said that she was more resistant to possible anxiety than her family, whom she described as prone to hypochondria:

Interviewer: And what made you decide to [use the app] for [your daughter] and not with her?

Interviewee: That I wanted to check it out first...My husband is a bit of a hypochondriac and so is my child. So when I sort of poke them, or it could be something dramatic, then I always don’t know what will come of it. That’s why I did a pre-check for myself first.

NT21

Health system accessibility or formal requirements because of their work further influenced users’ motivation to take health-related actions:

Interview partner: ...It...is also one of the reasons why you don’t like going to the doctor so much, is that it is hard to get an appointment with such specific doctors somehow. Especially a dermatologist or something like that, you have to wait two or three months and then you usually let it drop.

Interviewer: So access to a doctor or to care plays a role for you?

Interview partner: Yes. Yes. I think if I knew, okay, I’m going to get an appointment in one, two, three weeks, then I’d be more likely to have something like that checked out.

NT41

### Volition: Intention Formation (Preactional)

The motivational process described previously led to a planning stage for HRB during which users purposefully engaged with symptom checker apps with 3 distinct goals in mind. They sought to (1) understand and validate their condition, (2) receive recommendations for further action, and (3) communicate and document health-related issues.

#### Understanding and Validating One’s Condition

Users were interested in understanding their condition. The app provided an opportunity to find more information. Information seeking was described as one way to deal with the concerns:

...I see [the app] as a first aid, first source of information, where you can think about...: What do I do now? Then I can take a look or maybe I think again: What do I do now?...so as...again to update the normal knowledge that you have as a non-medical person.

NT37

Finding a clear cause also drove the users and induced HRB:

I woke up with 39 and something degrees. And of course I opened the app again and entered my symptoms, just to see...what it was saying now...Yes, then I knew...Okay, these are clearly the symptoms. Which of these is now Corona, which is maybe something else. Yes, I can still remember...that I was lying in bed suffering and had the app open.

NT05

Sometimes, users described that an element of surprise was needed to find an explanation for their concerns. They admitted that they were not aware of all possible causes of their conditions. The ability to be surprised was attributed to both physicians and the app as external information sources:

Maybe it’s (what is needed to accept a diagnosis) a “click” moment that you didn’t realize before. As an example, maybe a doctor or a diagnosis app asks a question [like: Does it get worse when it’s cold] and then you think to yourself: Ah, yes, when I think about it, it does get worse when it’s cold, or whatever.

NT24

Other times, users already had a suspicion of their own, which could be verified by the symptom checker app:

...I also have migraines and sometimes I can’t quite identify whether it’s a migraine or a headache. And the app often helps me and then I can practically distinguish: OK, do I treat it like a headache now or do I treat it like a migraine now.

NT41

In summary, the purpose of gathering information was to add to one’s own knowledge about the condition and actively seek out validation of one’s subjective impression.

#### Receiving Recommendations for Further Action

In addition to collecting more information about one’s condition, users stated wanting to receive options for further health-related actions to improve their condition:

What were the first experiences?...I used the feature best or most, simply these suggestions about which therapies you can take for which symptoms. And so I always looked here again to see what the app actually recommends.

NT44

Even if the app was primarily used for information gathering, the recommendations were received and considered:

...I looked to see what information was available if I somehow...well, with children there’s always something. And then I just thought the app was so good, because sometimes they had a few other tips. And then I just looked to see what one says, what the other says...It was always a push in the direction: “I should go to the doctor.”

NT21

Users also typed in symptom combinations several times to see whether anything changed in the app’s output:

Well, of course I also had accompanying symptoms during the heart rhythm disturbances. So sometimes trembling or high blood pressure, but some of it was only temporary. I then sort of started to play with that. I added the symptom once and indicated it and left it out once to see what it recommended. Because the option of selecting that a symptom only occurs temporarily or is concomitant, only temporarily, is not available in the app. And then I wanted to play around with what would come out in the end if I indicated this and didn’t indicate it. But apart from that, the reason was...I gave everything I had and always answered the questions truthfully.

NT19

If the sense of medical urgency was very high or life-threatening, app use was explicitly avoided. One participant described such a hypothetical scenario:

If I somehow had symptoms of a heart attack or something like that, then I would probably call the ambulance service directly and not look on the app to see if I really had a heart attack or if I was bleeding to death or something like that.

NT49

#### Communicating and Documenting Health-Related Issues

Users documented their own symptoms on the app:

It was recommended to me a long time ago to track symptoms, just to keep a diary of symptoms...

NT31

The app was also used to prepare for a visit to health care professionals. In that context, the documentation contained on the app was primarily used for the orderly and rapid presentation of concerns:

So I can already describe my state of health more specifically than if I hadn’t inquired beforehand.

NT06

### Volition: Intention Implementation

#### Personal Resources

To implement the purpose of the initiated action, users integrated personal, social, and digital resources into their behavior. One example leading to a health care visit was provided by one of the interview partners:

My doctor didn’t tell me that [oral iron supplement pills] could have side effects. And then I had discolored feces and abdominal pain and all sorts of things for a week or two...So I used the app and then I googled it and then I think I also talked to a friend about it. (00:04:27) She was like: Hey, maybe it’s from the iron tablets. And then I realized that it was the iron tablets. And then I stopped taking them and then I talked to my internal medicine physician and she was like: Yes...And prescribed me other iron tablets.

NT41

Personal resources were provided by biographical reference points such as previous experiences or special knowledge from one’s own professional activity or skills. As soon as the purpose of the action was determined in the preactional phase, personal resources were factored into the user’s action. Users described certain skills that they used in the process but also a sense of self-efficacy. If enough personal resources were available, users could actively decide against the app’s recommendation, as the following example shows:

And then I entered that [the symptoms]. The app said it could be a torn ligament or a fracture and that I should go to the emergency room. And then I thought: ...I don’t know how I could have broken any bone in the way I was walking. For me, the ligaments were more plausible...I’ve had it before, so I already had a bit of experience of what it’s like. And then I thought: OK, the app says one thing, but I’ll just look at it again in two or three days and see how it develops.

NT42

#### Social Resources

Users also intentionally and purposefully sought out social resources. Friends and family were assigned a role in understanding and responding to concerns:

...I once suspected a stroke and then I was at home and had a reduced field of vision and thought: What is this? But that was before I knew about these apps. And of course I immediately called a friend and said: What could this be? What is that? And she said: Make sure you have it checked out the next day.

NT21

Health care professionals such as physicians were assigned a special role—a visit to a health care professional was seen as mandatory to obtain a final validated diagnosis or to obtain health care services such as an authorization for sick leave or a prescription. The interactions with physicians were mostly intentionally initiated by the users if other resources were depleted or did not improve their situation:

And I had then already contacted my family doctor anyway, just from the fact that I also need a sick note...Of course, if this [the symptoms] were to last longer, the app would be of no use to me in the end, then I would have to go back to the neurologist or...the family doctor.

NT39

The only social resource with physical access to the users’ body were physicians as they could conduct physical examinations:

And then I went there [to the General Practitioner], described it again and showed it. Then...[she] turned the [painful arm] in different directions and said that no part, neither the forearm nor the upper arm muscle, is affected in any way. That comes in any case, exactly, so from the tendons...And then I just said: yes, [the concern comes] from climbing...And then she said: how much do you climb? So if you don’t climb four hours a day, it’s very unlikely that it comes from that.

NT31

Reviewing the input and results together with health care professionals (eg, a general practitioner [GP]) was mostly described hypothetically. However, the prospect of discussing app results with their GP was seen as beneficial by the users:

...what I would like is when you come to the doctor and say: I assume that I have this because I googled it or found out on an app. That he then says: Yes, what symptoms did you enter? Or: How did you then come up with this result?...So that he then, I don’t know, doesn’t have to start again with Adam and Eve, but that he can already inquire more intensively.

NT06

Mostly, users made a conscious decision to leave the app out of the social context of health care. They expected it to have a negative impact on patient-physician relationships:

Interviewer: Did you also mention there (at the GP) that you used the app?

Interview partner: No, because I have the impression that doctors tend to react badly to this. Because then, they always like to say Dr. Google...and the topic has a bit of a bad reputation.

NT07

#### Digital and Technological Resources

The app and search engines were seen as competitors in the same category with distinct advantages and challenges. Search engines were mainly used as a supplement to obtain information that was missing on the app:

...when you’re constantly googling, sometimes very worrying answers come up and you can also enter a lot of specific things in the app and it’s just more practical and you somehow feel, how should I put it, the diagnosis somehow feels more trustworthy than through Google.

NT41

...I missed in the symptom checkers, that you somehow get such a tip, such old home recipes...belly compress or something, and then such a guide to it...I then thought: ...now I’m googling, what can one do, yes, perhaps with a homeopathic approach. Because that’s what I was missing.

NT21

In contrast to app use, users found search engines overwhelming when they tried to check their symptoms there:

[Referring to searching concerns with internet search engines] In principle I never really figured it out, because there were 10, 15, 20 possibilities, and I couldn’t really assess it at all. Really.

NT19

There are other technological resources and devices such as users’ wearable devices or imaging procedures at the hospital in addition to the app. Devices able to monitor and measure bodily functions were described as determining and important factors that can contribute to a decision:

I...always measured the blood pressure. And even my [smartwatch], which is so clever, then recognized that there is a heart rhythm disturbance, at times. That time, it showed a rhythm disturbance...Then I thought: Okay, now I have to go to the doctor.

NT19

The user experience and usability features of the app were discussed. For example, the chatbot-based approach with questions asked to be answered by the user was perceived as both helpful and limited. Users missed the opportunity to clarify their input, ask questions, or address their own uncertainty and ambiguity:

...I would have put it (a surprising app-result) down to the fact that the app didn’t really understand me...because I somehow couldn’t really convey exactly what I wanted to say on the basis of the questions and the selection options...I would have said: OK, it just misunderstood me.

NT37

According to the users, affinity for technology played a role in the use of the app:

Yes, for my mum, for example, I use it (the app) quite often, because she’s not that into the internet and apps and stuff.

NT41

### Evaluation

The outcomes of the interaction between users and the symptom checker app were integrated into their subsequent experiences and interpreted in the context of their past health-related experiences. Once the HRB had been executed, a comparison was made as to whether the intention of the action had been fulfilled. Whether the app’s results represented an actual diagnosis was negotiated individually by each user. Users described having difficulty accepting the app results depending on the topic and their own interpretation of the results. If the 2 diverged too drastically, they were at a loss and saw potential problems in understanding or misinterpreting the given recommendations:

I think the app always told me: stress and mild depression or something like that. I didn’t feel that stressed, but yes, I think that was the thing...I read through it, but then I thought: Well, I still don’t know exactly what’s going on.

NT37

Concerning recommendations especially, users critically evaluated the app’s output. How they reacted to it depended on the intended purpose of the action, the user, and the context in which the assessment took place:

I was a bit shocked that [the app] now sends someone directly to the emergency room (with the app result tonsillitis). Because I work for a health insurance company...and I find it a bit exaggerated that patients are sent so quickly to the emergency room. They are so overloaded at the moment.

NT39

Users reported that they were reassured by validated external information about their condition. The app sometimes could provide such reassurance:

It’s a bit like this: Is it true now? Of course, we are aware, especially with this vaccination, that the side effects come from the vaccination. But somehow it’s still like that: Yes, you feel safer and somehow more confirmed, even if you only have it in an app, but, yes, you are somehow more reassured.

NT41

On the other hand, users also found the app results unsettling and misleading:

So it could really only be overstretched ligaments (user’s suspicion), this fracture (app result) made me a bit...A bit scared at that moment.

NT42

Users acknowledged that having received information contributed to a learning process that affected how they reacted to future symptoms:

I still have it on my mobile phone, I still like to look at it once in a while, even if it’s just to advance my knowledge a bit. And, yes, it has become...I have found that I look at it a little less, but also because I find that when things repeat themselves in some way, you already know how you could react.

NT44

## Discussion

### Principal Findings

#### Layperson Perspectives on Using Symptom Checkers for Health-Related Concerns

This study’s findings indicate that the use of a symptom checker app by layperson users is a multifaceted and iterative learning process that involves individual motivational factors; contextual elements; and interaction with digital, social, and personal resources. Both eHL and HL play a significant role in this process. The subsequent Discussion section addresses the 3 research questions.

The use of symptom checkers can be mapped onto the 4 distinct steps of the Rubicon model: motivation, intention formation, intention implementation, and evaluation [48]. These stages can be viewed as an iterative learning cycle that generates health-related experiences according to the constructivist paradigm [44]. During the motivational stage, users negotiate app use depending on the context. If users choose to use the app, they do so intentionally for 3 distinct purposes: obtaining information and health information–seeking behavior (HISB), receiving recommendations, and documenting and communicating health-related issues. The purpose determines the user’s strategy and the resources used to achieve it, including personal, social, and digital or technological resources. Users’ experiences with the symptom checker app can influence their motivation to use it in the future and how they incorporate it into their HRB. Making meaning plays a crucial role in this as users try to understand their bodily perceptions and symptoms [43]. This process aligns with the model proposed by von Wagner et al [29], which links HL and HRB and emphasizes the role of learning in HL. Our study highlights that the use and evaluation of symptom checker apps in line with the dynamic concept of HL varies over time and is dependent on the context and purpose of use [25,27,28,49]. This poses a challenge when researching symptom checker apps [4,7,38].

#### Entry Points and Possible Outcomes of the Symptom Checker App Use Process

##### Entry Points

Entry points were identified during the motivational or predecisional phase. They were influenced by the context in which symptom checker app use was considered [29]—in addition to the user’s own health needs, situations involving the health needs of others were also relevant. Motivational determinants included attentiveness toward symptoms, past experiences (biographical factors), confidence in one’s health, trust in the app, and one’s ability to respond to symptoms. Some of these factors for app use were also identified by Aboueid et al [4,6] and Meyer et al [7]. The accessibility of health care and availability of social networks were described as external factors. Aboueid et al [4] identified the lack of accessibility of health care as an enabler of app use, whereas social influences were identified as a barrier. The diary study from the CHECK.APP project found that the first occurrence of a symptom and certain symptoms such as heart-, skin-, or eye-related concerns increased the likelihood of symptom checker app use [16]. In the interview sample, interview partners considered the aforementioned factors in their decision to use the app but did not specify whether they were facilitators or enablers. Although many of the listed factors have been identified in previous research, their role in symptom checker app use is ambiguous as they can act as both barriers and enablers [3,10,21]. As both situational and individual factors shape when and for what purpose symptom checker apps are used, the term “context factors” seems much more appropriate than “barriers and enablers” [3,10,27,28].

##### Outcomes

###### Meaningful Learning Experiences

The most common outcome of using symptom checker apps as a digital information source and HISB is the act of learning, which is difficult to measure [1]. This outcome is expressed through the purpose of understanding one’s condition based on users’ need for more information. Users already have predefined concepts about their condition in mind when planning their course of action. Therefore, they also want to validate these concepts by gathering information. In a substudy of the CHECK.APP project by Wetzel et al [9], it was found that symptom checker apps were the least important for HISB in the general population. The subsample of active symptom checker app users reported that internet use and consulting a physician were more important for HISB than symptom checker apps [9]. In the study by Meyer et al [7], most symptom checker app users stated that they used symptom checker apps to understand the cause of their condition and found the information they received useful. During the evaluation stage, users consider the extent to which the information is meaningful. They base their evaluation on their experiences with symptom checker app use and the health-related actions and determine whether the intended purpose was fulfilled and whether meaningful learning experiences were generated [24,44].

###### HRB Outcome

Another type of outcome is HRB, which is planned in the intention formation stage and implemented in the following stage. The actions described in the intention implementation stage relate to measurable HRB, such as visiting a health care professional or seeking advice from friends or family [29]. Depending on the purpose of app use, 2 types of behavior can be distinguished in this category.

As a result of using symptom checker apps for the purpose of receiving recommendations for further action, such recommendations are generated. This occurs frequently—in the sample of Meyer et al [7], this purpose ranked second among symptom checker app users.

Our interview partners considered whether to follow app recommendations thoroughly. If recommendations do not align with users’ concepts and expectations, they are sometimes discarded as unrealistic, effectively preventing health care visits. This mismatch between concept and recommendation can also cause anxiety, creating the need for more information or confirmation by health care professionals [10,11,16]. Turner et al [50] also observed the rejection of app recommendations, especially if they were given in a context in which the action could not be implemented (eg, out of hours). Verzantvoort et al [24] found that users intended to follow app advice if the advice was to contact their GP during the daytime (75%) and practice self-care (67%). If the concept and recommendation align or if users are very anxious or surprised about the result, a visit to health care professionals is a possible outcome.

To better understand this behavior, a dialogue option could be implemented in symptom checker apps asking the following—“Have you followed my recommendation?”—and whether further resources such as health care professionals have been contacted. That way, not only symptoms but also user behavior and impact of the symptom checker app on health care use could be traced.

Communicating and documenting health-related information serves users’ need to structure their experiences and prepare for contact with health care services. This sets up symptom checker apps as a possible interface between the user and health care services. This purpose has not been described yet apart from the CHECK.APP project [10]. Symptom checker apps can increase patient autonomy, as other research has postulated [17]. However, users specifically omit telling their physicians about symptom checker app use.

According to our sample, direct communication with health care professionals over app results and app use was avoided. Aboueid et al [4] found that attitudes toward physicians and the health care system may impact app use. Meyer et al [7] reported that only approximately 50% of patients using symptom checker apps considered informing their physicians about their symptom checker app use. The study indicates that users may share the output of the app, including given recommendations or probable diagnoses. However, they are less likely to inform their physicians that they used a symptom checker app to generate these results. Further research is needed to explore ways to facilitate open communication between health care professionals and symptom checker app users. The quality of the communication and its contents are suggested as measures for this outcome.

###### Satisfaction With App Use

A third outcome is user satisfaction with the app, which emerges during the evaluation stage. This outcome is closely linked to the app’s intended purpose and the 2 previously mentioned outcomes: HRB and learning. As such, satisfaction with symptom checker apps represents a multifaceted and complex construct [13,24].

Satisfaction resulting from app use depends on how well the users perceive the symptom checker app’s alignment with their needs, expectations, and intended purpose. Users become insecure and sometimes dissatisfied with the app when their concept does not match the output. This association with user satisfaction indicates that users’ expectations also play a role in how they validate the app [24]. In a substudy of the CHECK.APP project, Müller et al [10] distinguished between user expectations and motivations for app use and found that these factors play a role in symptom checker app use. In fact, according to the sample in the study Kopka et al [51], 33% of symptom checker app users reported that the symptom checker app’s usefulness varied. Turner et al [50] conducted a study on an online symptom checker and found that users were dissatisfied with it because too little information on what to do themselves was provided. From their point of view, the outcome did not fit their intended purpose. This should be critical for first-time users but can also influence how users validate the app over time [51].

This indicates that simply measuring a single outcome of app use may be a shortcoming of previous research as it ignores the context of use and the intended purpose. Symptom checker apps provide a wide range of potential services that are integrated into a complex network of resources [4,5,7,13,18,20]. Therefore, researchers and developers must consider addressing the intended purpose of use and expectations and specify the desired outcome.

#### HL and eHL in Symptom Checker Use

##### Motivational Stage

The main finding of this study was that both eHL and HL are present throughout every stage of the complex process of symptom checker app use. However, different aspects of these concepts are relevant for different stages. Therefore, we will discuss the expression of eHL and HL in relation to the stages of the symptom checker app use process in the following sections.

In the motivational stage, users are driven primarily by the presence of a health-related concern and the context in which it occurs. Personal resources such as the ability to process health information and access services (as defined by the concept of HL [25]) or to navigate electronic, digital, and mobile health information (as defined by eHL [1]) play a crucial role. However, the application of these skills is influenced and shaped by the surrounding context [27,28]. The description of these skills by the interview partners can also be conceptualized as self-efficacy and self-care, which are interlinked with HL [52]. In a substudy of the CHECK.APP project, Wetzel et al [8] found that, unlike eHL, self-efficacy could be a determinant of app use—however, a sensitivity analysis revealed that the initial correlation was not viable. Kopka et al [51] found that individuals who perceived symptom checker apps as useful had higher self-efficacy. This ambiguity can be resolved by considering the context of use as an additional factor. Some symptoms may be more alarming than others and may exceed the available skills of even those with high HL [16].

Trust in apps, or the lack thereof, plays a role at the motivational stage. Regarding eHL, the interview partners in our sample positioned themselves as having high technical knowledge and the ability to critically appraise app output (eHL). They trusted the app but also their HL skills. This is consistent with the observation made by Kopka et al [11]: users with high eHL are more likely to trust symptom checker apps, possibly because they have higher confidence in their ability to critically evaluate app output. Conversely, lack of trust has been identified as a factor in intentional nonuse [9]. Neither technology affinity nor eHL are predictors that differentiate symptom checker app users from nonusers [8]. Aboueid et al [14] identified different technology affinity profiles as predictors of future app use for self-triage. Both technology affinity and trust represent attitudes rather than skills. They shape expectations and motivation to use apps but are not predictors [10].

In our sample, interview participants did not link their attitudes toward the app with the frequency of its use. Instead, their motivation was shaped by specific and meaningful experiences, such as situations in which the app provided valuable assistance with a critical symptom, subsequently confirmed by a health care professional. Research suggests that factors such as hypochondria [8] and the presence of new or unfamiliar symptoms [16] can drive app use, indicating that eHL and HL influence not only the frequency of app use but also the purposes for which symptom checker apps are used and the manner in which they are used. On the basis of these findings, we conclude that frequency of app use is an oversimplified parameter and should be interpreted with caution in future analyses.

In summary, the skill sets defined in HL and eHL, as well as context factors, influence how the motivational stage unfolds [27,28].

##### Intention Formation

Symptom checker apps can serve as both an information source and a tool for shaping one’s HRB. Navigating digital tools for information gathering and evaluating this information is related to eHL. Applying the information to one’s behavior and learning from it is more closely related to HL [24,29,30]. Therefore, planning to use symptom checker apps relates to both eHL and HL [1,25].

Users with high HL may anticipate the app’s output and choose not to use it or only use it for information gathering and HISB. In addition, users may experiment with different inputs to observe changes in the output. This demonstrates the interconnection between HL and eHL during the intention formation stage. Users understand the inner workings of the app (eHL) and plan to use it to satisfy their information needs but do not intend to actually follow its advice (HL) [1,53]. According to Kopka et al [11], users are more easily persuaded to follow app recommendations when they are ambivalent or unsure about their own decision. This aligns with interview partners’ self-reports that they were less inclined to follow the app’s recommendations if they felt that their condition was critical or if the recommendation significantly differed from their own concept (eg, recommendation to seek emergency help when users felt perfectly healthy). In addition, using apps to communicate about symptoms connects the concepts of eHL and HL with a social context in health care [2,26].

##### Intention Implementation

Users use a range of resources associated with eHL and HL to achieve their intended goals. Key personal resources include the ability to evaluate one’s health and navigate the health care system (HL), critically assess information from various platforms, and effectively use and understand digital applications (eHL). These resources are pivotal in shaping how symptom checker apps are integrated into users’ actions [1,25,28,49]. In our sample, users expressed confidence in their eHL skills, including media literacy, information literacy, and computer literacy. However, the extent to which they used these skills depended on their purpose and the availability of other resources.

The demand for applying interactive HL is not met by symptom checker apps because they do not allow for direct communication with others in a social context. This is particularly evident when users seek to validate their own ideas about their condition or receive recommendations for action. In such cases, users may seek out social resources and discuss their condition with friends and family. Alternatively, they may present the app results to a health care professional without disclosing their use of the app. This demonstrates that users extend app use to other contexts, integrating those contexts into their overall user experience [29,48]. This study demonstrated that HRB can transition from digital interaction to social interaction in the context of symptom checker app use. This finding aligns with the concept of distributed HL by Edwards et al [34], highlighting the importance of social networks in realizing the benefits of symptom checker apps. Real-life interaction can provide what the app interaction lacks. The ability to actively listen, notice, and respond to emotional nuances; provide support; and express understanding for the person’s situation is also important. According to the observation by Wetzel et al [9], symptom checker app users still refer to friends, family, and physicians for HISB, albeit to a lesser extent than nonusers. Future research in ethics and social sciences will reveal the impact of large language models that mimic empathic responses and conversations on the use of symptom checker apps. Preliminary data suggest that such models may enhance the symptom checker app experience [54].

The interview partners considered symptom checker apps as one of the many digital resources available today. According to users, the app asked dichotomous questions, leaving them with a feeling of not being able to tell their whole story, address ambiguity, or ask questions themselves [24]. However, symptom checker apps offer a more personalized experience than internet searches [4], which remain a core component of HISB even among symptom checker app users [9]. The personal resources that enable users to use the app and apply its results also impact the integration of other digital resources into their HRB [1]. Therefore, there is potential in enhancing interoperability between different technologies such as wearables and apps.

##### Evaluation

The app’s results, whether they provide information or recommendations, may challenge users’ preexisting concepts regarding health-related issues. The extent to which users follow these recommendations depends on their preconceived notions about their condition; their expectations for its resolution; and their ability to cope with uncertainty, which is related to HL [10,25].

Users evaluate whether the app results align with the severity of their condition, their expectations of the appropriate course of action, and past experiences. This evaluation requires critical HL [25]. If users feel uncertain due to app use, they may need to contact others, such as health care professionals or their social network, for additional validation outside the app. Individuals with high levels of hypochondria [8] or anxiety [51] may be considered vulnerable groups in this context, particularly if they have low self-efficacy and HL and lack access to social or health care resources.

For the interview partners, symptom checker apps served as an interface among information gathering [4,9], learning and sensemaking [7,43], and interaction with the health care system [9,22]. In summary, the skill set necessary for meaningful interaction with symptom checker apps in the context of health care services is best described using eHL and HL. Together with contextual factors, both models can provide a deeper understanding of the dynamic mechanisms underlying symptom checker app use.

### Implications for Health Care and Research With Regard to Symptom Checker App Use

Overreliance on users’ HL and eHL results in wasted potential [6]. The findings of this study support the idea that, for the implementation of symptom checker apps in health care, transparency is crucial [7,12]. The lack of transparency and reliance on users’ HL and eHL are design flaws in symptom checker apps, which, from a legal standpoint, only present information to be interpreted by the user who provided the input. In a worst-case scenario, users may feel isolated and insecure [3,21]. If the app was designed as a communication tool between physicians and users, such as in teleconsultations, their potential as symptom trackers and decision aids for both user groups could be realized in a safe setting. Users’ insecurities could be discussed with a health professional, and possible harm, such as health anxiety, could be reduced.

The skill sets provided by eHL and HL might be the reason why users are able to use symptom checker apps to their health benefit at all despite symptom checker apps’ questionable diagnostic accuracy [18,38,55]. Concrete and meaningful experiences with the app shaped users’ attitudes toward and evaluation of the app. A pure cause-and-effect relationship, as suggested by its user interface principle (input–algorithm or artificial intelligence–output), is prevented by users’ HL, self-efficacy, and self-care. Satisfaction with app use is influenced by users’ HL, context, and experiences [10,13,24,29]. Symptom checker app development and research should take into account user expectations, context of use, and the need for social interaction.

Our study uncovered phenomena that require further investigation in the future. These include symptom checker app use in various social settings [24]. Our results show that users frequently engage with symptom checker apps not only for their own health concerns but also to assess symptoms of family members and friends. This social use—such as evaluating a partner’s symptoms or a parent using the app for their child—occurs more often than previously reported in the literature. Different technological resources such as wearables, other apps, and internet searches were perceived as possible contributors to the 3 purposes by the interview partners. Thus, research on the topic should consider the entire landscape of digital resources and their interoperability.

### Limitations

Experience-based research is limited to the information that interview participants are willing to share about themselves. It is not possible to directly observe the psychological processes that occur in someone’s mind. However, by collecting and synthesizing users’ stories and perspectives, we were able to describe a general process. The Rubicon model helped us organize the stories coherently and with a focus on motivation.

It is important to critically evaluate the method and sample used. It should be noted that, even in our sample of predominantly younger, White, well-educated female users, each individual had their own perspective on how the app fulfilled its purpose in the described contexts. While a general process could be derived, the outcomes were highly dependent on the user. In addition, the results are primarily applicable to the context of the German health care system and, therefore, can only be transferred to similar health care systems, which is a common challenge in symptom checker app research [38].

Our sample consisted of predominantly very reflective individuals with presumably high HL. Our sampling strategy to approach users based on diverse characteristics could only partly be implemented because the sample from which we recruited the interview partners represented the current major user group of symptom checker apps: young, female, and well educated individuals [5,9,17]. Thus, the sample reflects the most typical characteristics of symptom checker app users, indicating that the results are likely applicable to this core group [3,9].

While the mixed methods approach of the CHECK.APP project allowed for the description of this population from different perspectives using different methods, it limits the applicability of our results to individuals with less HL. We can neither make assumptions about nor extend our findings to marginalized groups excluded from app use [15,56].

The integrative basic method builds on the tradition of interpretative-reconstructive qualitative methods such as ethnomethodological conversation analysis and narrative analysis. It proved to be suitable to analyze how medical laypersons make sense of the symptom checker app. While we could show in another study of the CHECK.APP project how GPs position symptom checker apps in the black box of the “unorganized stage” of patients’ reflections on their symptoms [40], our study succeeded in shedding light on this exact black box.

Another limitation of this study is the focus on just 1 app. While we believe to have extrapolated generalizable information, it is possible that other apps may yield different user experiences and human-app interactions. In addition, due to the pandemic, the interviews were conducted on the web via videoconferencing software. It is possible that conducting in-person interviews would have attracted different interviewees and revealed additional information due to the different setting.

While software-supported tools such as MAXQDA or f4analyse exist to support qualitative analysis and the authors and analysts are well acquainted with them, we deliberately chose not to use these tools especially for the interpretative parts of the analysis because their workflow is optimized for categorization of qualitative data, not so much for in-depth interpretative analyses. To ensure that our operationalization remained understandable, we provided data examples in the results text.

### Conclusions

In our qualitative interview study, we could demonstrate that, from symptom checker app users’ perspective, a simple cause-and-effect relationship between symptoms and symptom checker app use is unlikely, at least in individuals with high eHL and HL. Rather, symptom checker app use is described as a complex, cyclic process. Context-dependent and biographical factors, as well as the dynamic concepts of eHL and HL, were expressed in users’ descriptions.

According to our limited sample, symptom checker apps are used for 3 distinct purposes: understanding one’s condition, receiving recommendations for action, and communicating on and documenting health-related information. Each purpose warrants its own planning, implementation, and evaluation in HRB. Each purpose may be implemented using different personal, social, and technological resources.

Symptom checker apps have shortcomings with respect needs related to interactive HL. Users seek external validation of app findings in their social networks and professional health care services. However, symptom checker apps have the potential to become an interface between users and health care services. This potential has not been realized, which should be a design goal for their continued development.

## Appendix

Multimedia Appendix 1 Standards for Reporting Qualitative Research statement checklist.

Multimedia Appendix 2 Interview guide.

## Abbreviations

CHECK.APP: Ethical, Legal, and Social Implications of Symptom Checker Apps in Primary Health Care
eHL: eHealth literacy
GP: general practitioner
HISB: health information–seeking behavior
HL: health literacy
HRB: health-related behavior

## Data Availability

The datasets generated or analyzed during this study are available from the corresponding author on reasonable request. Due to data protection regulations, interview data can only be made available if the study participant associated with the data approves the request.

## References

[1] Norman CD, Skinner HA (2006). eHealth literacy: essential skills for consumer health in a networked world. J Med Internet Res.

[2] Levin-Zamir D, Bertschi I (2018). Media health literacy, eHealth literacy, and the role of the social environment in context. Int J Environ Res Public Health.

[3] Müller R, Klemmt M, Ehni HJ, Henking T, Kuhnmünch A, Preiser C, Koch R, Ranisch R (2022). Ethical, legal, and social aspects of symptom checker applications: a scoping review. Med Health Care Philos.

[4] Aboueid S, Meyer S, Wallace JR, Mahajan S, Chaurasia A (2021). Young adults' perspectives on the use of symptom checkers for self-triage and self-diagnosis: qualitative study. JMIR Public Health Surveill.

[5] Aboueid S, Liu RH, Desta BN, Chaurasia A, Ebrahim S (2019). The use of artificially intelligent self-diagnosing digital platforms by the general public: scoping review. JMIR Med Inform.

[6] Aboueid S, Meyer SB, Wallace JR, Mahajan S, Nur T, Chaurasia A (2021). Use of symptom checkers for COVID-19-related symptoms among university students: a qualitative study. BMJ Innov.

[7] Meyer AN, Giardina TD, Spitzmueller C, Shahid U, Scott TM, Singh H (2020). Patient perspectives on the usefulness of an artificial intelligence-assisted symptom checker: cross-sectional survey study. J Med Internet Res.

[8] Wetzel AJ, Klemmt M, Müller R, Rieger MA, Joos S, Koch R (2024). Only the anxious ones? Identifying characteristics of symptom checker app users: a cross-sectional survey. BMC Med Inform Decis Mak.

[9] Wetzel AJ, Koch R, Koch N, Klemmt M, Müller R, Preiser C, Rieger M, Rösel I, Ranisch R, Ehni HJ, Joos S (2024). 'Better see a doctor?' Status quo of symptom checker apps in Germany: a cross-sectional survey with a mixed-methods design (CHECK.APP). Digit Health.

[10] Müller R, Klemmt M, Koch R, Ehni HJ, Henking T, Langmann E, Wiesing U, Ranisch R (2024). "That's just Future Medicine" - a qualitative study on users' experiences of symptom checker apps. BMC Med Ethics.

[11] Kopka M, Schmieding ML, Rieger T, Roesler E, Balzer F, Feufel MA (2022). Determinants of laypersons' trust in medical decision aids: randomized controlled trial. JMIR Hum Factors.

[12] Miller S, Gilbert S, Virani V, Wicks P (2020). Patients' utilization and perception of an artificial intelligence-based symptom assessment and advice technology in a British primary care waiting room: exploratory pilot study. JMIR Hum Factors.

[13] Chambers D, Cantrell AJ, Johnson M, Preston L, Baxter SK, Booth A, Turner J (2019). Digital and online symptom checkers and health assessment/triage services for urgent health problems: systematic review. BMJ Open.

[14] Aboueid S, Meyer SB, Wallace J, Chaurasia A (2021). Latent classes associated with the intention to use a symptom checker for self-triage. PLoS One.

[15] Luger TM, Houston TK, Suls J (2014). Older adult experience of online diagnosis: results from a scenario-based think-aloud protocol. J Med Internet Res.

[16] Wetzel AJ, Preiser C, Müller R, Joos S, Koch R, Henking T, Haumann H (2024). Unveiling usage patterns and explaining usage of symptom checker apps: explorative longitudinal mixed methods study. J Med Internet Res.

[17] Arellano Carmona K, Chittamuru D, Kravitz RL, Ramondt S, Ramírez AS (2022). Health information seeking from an intelligent web-based symptom checker: cross-sectional questionnaire study. J Med Internet Res.

[18] Wallace W, Chan C, Chidambaram S, Hanna L, Iqbal FM, Acharya A, Normahani P, Ashrafian H, Markar SR, Sounderajah V, Darzi A (2022). The diagnostic and triage accuracy of digital and online symptom checker tools: a systematic review. NPJ Digit Med.

[19] Schmieding ML, Kopka M, Schmidt K, Schulz-Niethammer S, Balzer F, Feufel MA (2022). Triage accuracy of symptom checker apps: 5-year follow-up evaluation. J Med Internet Res.

[20] Semigran HL, Linder JA, Gidengil C, Mehrotra A (2015). Evaluation of symptom checkers for self diagnosis and triage: audit study. BMJ.

[21] Pairon A, Philips H, Verhoeven V (2022). A scoping review on the use and usefulness of online symptom checkers and triage systems: how to proceed?. Front Med (Lausanne).

[22] Ceney A, Tolond S, Glowinski A, Marks B, Swift S, Palser T (2021). Accuracy of online symptom checkers and the potential impact on service utilisation. PLoS One.

[23] Knitza J, Muehlensiepen F, Ignatyev Y, Fuchs F, Mohn J, Simon D, Kleyer A, Fagni F, Boeltz S, Morf H, Bergmann C, Labinsky H, Vorbrüggen W, Ramming A, Distler JH, Bartz-Bazzanella P, Vuillerme N, Schett G, Welcker M, Hueber AJ (2022). Patient's perception of digital symptom assessment technologies in rheumatology: results from a multicentre study. Front Public Health.

[24] Verzantvoort NC, Teunis T, Verheij TJ, van der Velden AW (2018). Self-triage for acute primary care via a smartphone application: practical, safe and efficient?. PLoS One.

[25] Nutbeam D (2000). Health literacy as a public health goal: a challenge for contemporary health education and communication strategies into the 21st century. Health Promot Int.

[26] Nutbeam D, Lloyd JE (2021). Understanding and responding to health literacy as a social determinant of health. Annu Rev Public Health.

[27] Parker RM, Ratzan S (2019). Re-enforce, not re-define health literacy-moving forward with health literacy 2.0. J Health Commun.

[28] Parker R, Ratzan SC (2010). Health literacy: a second decade of distinction for Americans. J Health Commun.

[29] von Wagner C, Steptoe A, Wolf MS, Wardle J (2009). Health literacy and health actions: a review and a framework from health psychology. Health Educ Behav.

[30] Neter E, Brainin E (2019). Association between health literacy, eHealth literacy, and health outcomes among patients with long-term conditions. Eur Psychol.

[31] Paasche-Orlow MK, Wolf MS (2007). The causal pathways linking health literacy to health outcomes. Am J Health Behav.

[32] Stormacq C, Van den Broucke S, Wosinski J (2019). Does health literacy mediate the relationship between socioeconomic status and health disparities? Integrative review. Health Promot Int.

[33] Stormacq C, Wosinski J, Boillat E, Van den Broucke S (2020). Effects of health literacy interventions on health-related outcomes in socioeconomically disadvantaged adults living in the community: a systematic review. JBI Evid Synth.

[34] Edwards M, Wood F, Davies M, Edwards A (2015). 'Distributed health literacy': longitudinal qualitative analysis of the roles of health literacy mediators and social networks of people living with a long-term health condition. Health Expect.

[35] Sørensen K, Pelikan JM, Röthlin F, Ganahl K, Slonska Z, Doyle G, Fullam J, Kondilis B, Agrafiotis D, Uiters E, Falcon M, Mensing M, Tchamov K, van den Broucke S, Brand H (2015). Health literacy in Europe: comparative results of the European health literacy survey (HLS-EU). Eur J Public Health.

[36] Berkman ND, Sheridan SL, Donahue KE, Halpern DJ, Crotty K (2011). Low health literacy and health outcomes: an updated systematic review. Ann Intern Med.

[37] Fiske A, Buyx A, Prainsack B (2020). The double-edged sword of digital self-care: physician perspectives from Northern Germany. Soc Sci Med.

[38] Ilicki J (2022). Challenges in evaluating the accuracy of AI-containing digital triage systems: a systematic review. PLoS One.

[39] Wetzel AJ, Koch R, Preiser C, Müller R, Klemmt M, Ranisch R, Ehni H, Wiesing U, Rieger MA, Henking T, Joos S (2022). Ethical, legal, and social implications of symptom checker apps in primary health care (CHECK.APP): protocol for an interdisciplinary mixed methods study. JMIR Res Protoc.

[40] Preiser C, Radionova N, Ög E, Koch R, Klemmt M, Müller R, Ranisch R, Joos S, Rieger MA (2024). The doctors, their patients, and the symptom checker app: qualitative interview study with general practitioners in Germany. JMIR Hum Factors.

[41] O'Brien BC, Harris IB, Beckman TJ, Reed DA, Cook DA (2014). Standards for reporting qualitative research: a synthesis of recommendations. Acad Med.

[42] Malterud K, Siersma VD, Guassora AD (2016). Sample size in qualitative interview studies: guided by information power. Qual Health Res.

[43] Bruner J (1990). Acts of Meaning.

[44] Kolb DA (1984). Experiential Learning: Experience as the Source of Learning and Development.

[45] Kruse J (2014). Qualitative Interviewforschung: Ein integrativer Ansatz.

[46] Davies B, Harré R (2007). Positioning: the discursive production of selves. J Theory Soc Behav.

[47] McAdams DP, Schwartz SJ, Luyckx K, Vignoles VL (2011). Narrative identity. Handbook of Identity Theory and Research.

[48] Achtziger A, Gollwitzer PM, Heckhausen J, Heckhausen H (2008). Motivation and volition in the course of action. Motivation and Action.

[49] Parker R (2009). Measuring health literacy: what? So what? Now what?. Measures of Health Literacy: Workshop Summary.

[50] Turner J, Knowles E, Simpson R, Sampson F, Dixon S, Long J, Bell-Gorrod H, Jacques R, Coster JE, Yang H, Nicholl J, Bath P, Fall D, Stone T (2021). Impact of NHS 111 Online on the NHS 111 telephone service and urgent care system: a mixed-methods study. Health Serv Deliv Res.

[51] Kopka M, Scatturin L, Napierala H, Fürstenau D, Feufel MA, Balzer F, Schmieding ML (2023). Characteristics of users and nonusers of symptom checkers in Germany: cross-sectional survey study. J Med Internet Res.

[52] Lee EH, Lee YW, Moon SH (2016). A structural equation model linking health literacy to self-efficacy, self-care activities, and health-related quality of life in patients with type 2 diabetes. Asian Nurs Res (Korean Soc Nurs Sci).

[53] Neter E, Brainin E (2012). eHealth literacy: extending the digital divide to the realm of health information. J Med Internet Res.

[54] Ayers JW, Poliak A, Dredze M, Leas EC, Zhu Z, Kelley JB, Faix DJ, Goodman AM, Longhurst CA, Hogarth M, Smith DM (2023). Comparing physician and artificial intelligence chatbot responses to patient questions posted to a public social media forum. JAMA Intern Med.

[55] Schmieding ML, Mörgeli R, Schmieding MA, Feufel MA, Balzer F (2021). Benchmarking triage capability of symptom checkers against that of medical laypersons: survey study. J Med Internet Res.

[56] Wang X, Luan W (2022). Research progress on digital health literacy of older adults: a scoping review. Front Public Health.

